# Meaningful Use of Electronic Health Records and Ambulatory Healthcare Quality Measures

**DOI:** 10.7759/cureus.13036

**Published:** 2021-01-31

**Authors:** Duaa Alammari, Jim E Banta, Huma Shah, Ellen Reibling, Majed Ramadan

**Affiliations:** 1 Health System Management, King Saud bin Abdulaziz University for Health Sciences, Riyadh, SAU; 2 Public Health, Loma Linda University, Loma Linda, USA; 3 Emergency Medicine, Loma Linda University, Loma Linda, USA

**Keywords:** electronic health records, quality measures, offices-based physician, national ambulatory medical care survey, screening, ambulatory care setting, meaningful use

## Abstract

Introduction

Electronic Health Record (EHR) adoption rates for office-based physicians doubled between 2008 and 2015, from 42% to 89%, and more than 60% of all office-based physicians achieved meaningful use by 2016. The US government has paid billions of dollars in incentives to promote EHR meaningful use. Nonetheless, evidence linking EHR meaningful use to quality measures improvements is limited.

Objective

This study aims to examine the relationship between EHR meaningful use and capabilities among four quality measures in an ambulatory healthcare setting.

Study design

A cross-sectional study design of the 2015-2016 National Ambulatory Medical Care Survey dataset.

Methods

We used adjusted multivariate regression models to examine associations between (a) EHR meaningful use and (b) 10 EHR-computerized capabilities, with four quality measures (blood pressure screening, tobacco use screening, obesity screening, and obesity education).

Results

We analyzed 30,787 office visits, representing an annual estimate of 680 million national office visits. Results showed that 95% of visits were to offices meeting EHR meaningful use criteria. We found one positive association between EHR meaningful use and obesity screening (OR= 3.5, 95% CI [1.742-6.917]). We also found eight positive associations between EHR capabilities and three quality measures (screening for blood pressure and obesity, and obesity education). These associations included five EHR-computerized capabilities: “record patient problem list”, “view lab results”, “Reminders for interventions/screening”, “Order lab results” and “Recording clinical notes”. No EHR capability was associated with screening for tobacco use.

Conclusions

We looked at a handful of screening-oriented quality measures in ambulatory healthcare and found limited associations with EHR meaningful use but multiple positively significant associations with EHR capabilities. Although EHR meaningful use has become more commonly used, offering substantial administrative efficiency over paper records, current patterns of EHR meaningful use do not always appear to translate into a better quality of care in physician offices. However, quality measures used represent limited procedures for a handful of specific conditions and not the overall healthcare aspect.

## Introduction

Health information technology use has grown rapidly nationwide [1]. Electronic health records (EHRs) are becoming a substantial element in the process of patient care delivery. The rates of EHR adoption doubled between 2008 and 2015, from 42% to 89%, at office-based physicians [2-3]. According to the National Electronic Health Record survey, 78% and 80% of office-based physicians reported using certified EHR systems in 2015 and 2017, respectively [2-3]. Certified EHR use began in 2011, when the Medicare and Medicaid EHR Incentive Programs were launched to motivate eligible physicians and hospitals to adopt, upgrade, and use certified EHR systems to demonstrate meaningful use [4-5]. Providers who did not successfully demonstrate meaningful use are subject to payment adjustments from Medicaid and Medicare as a penalty [4,6-7]. The US government has paid more than $24.8 billion in Medicare and $6 billion in Medicaid incentives to promote EHR use and meaningful use between 2011 and 2018 [8]. As a result, over 95% of eligible hospitals and 60% of all office-based physicians have achieved meaningful use in 2016 [1,3].

The Incentive Programs introduced meaningful use and its definitions in three stages. Stage 1 (2011-2015): certified EHR must be used in a meaningful manner to capture clinical data and store it in a structured format to improve patient outcomes; Stage 2 (2015-2017): to electronically exchange health information among healthcare providers and patients to promote interoperability; Stage 3 (2018 and beyond): to improve the Incentive Programs and simplify reporting requirements in addition to the previous requirements [4,6-7,9]. Eligible professionals report Clinical Quality Measures (CQMs) data to the Centers of Medicare and Medicaid Services (CMS) to track the quality of care services and demonstrate meaningful use in healthcare facilities. CQMs are tools that help measure and track the quality of health care services provided and can be electronically generated by EHRs [10]. As a result of the US government’s efforts, EHR adoption and its meaningful use have grown tremendously. Nonetheless, evidence linking national EHR meaningful use to quality measures improvements is limited [11-14].

Researchers found that using an EHR system that meets meaningful use was positively associated with a higher probability of reporting clinical benefits and overall patient care [15]. Also, a study that examined the association of using different levels of EHR systems and four quality measures compositely found that hospitals saw improvements in quality after transitioning to a system that can fulfill meaningful use standards [13]. On the other hand, studies indicated that there is no consistent benefit or difference in quality measures when using EHRs meeting meaningful use [11-12]. Additionally, Kern, Edwards, and Kaushal's 2016 results revealed that fewer lab tests, primary care visits, and emergency visits were needed for patients seen by physicians achieving EHR meaningful use when compared to physicians who did not achieve meaningful use [14].

While meaningful use has been studied with different quality measures, we did not find studies in previous literature focusing on the specific quality measures included in this research. Our study included four quality measures: screening for tobacco use, blood pressure screening, obesity screening, and education. These measures have been studied in previous literature with EHR use but not meaningful use. Researchers found the EHR use improved the quality of tobacco prevention and blood pressure screening [16-18] while obesity screening and education had inconsistent results [18-20].

EHR capabilities/functions

EHR functions or capabilities are computerized tools employed in the EHR system to be used by providers to enhance the process of patient care and promote safety. There are different types of capabilities such as detailed patient data (e.g., demographics, diagnoses, vital signs, allergies, laboratory results), and decision-support capabilities (e.g., the ability to alert providers to potential drug-drug interactions, prevention measures, procedures) [21].

Ancker et al. (2015) examined three groups of functionalities (electronic reminders, order, and view results) with 18 quality measures, including obesity screening (BMI), obesity education, tobacco use screening, and blood pressure management for diabetics. Results showed a statistically significantly higher performance in quality measures when functionalities were used during visits to physician offices [16]. Likewise, Samal et al. (2014) also found that a set of EHR functionalities including reminders, test results, order entry, visit notes, problem lists, and medication lists have been associated with higher quality blood pressure management [11]. Also, Poon et al. (2010) concluded that the availability and use of EHR functionalities were positively associated with higher quality for some measures [22]. These associations were mainly for recording problem lists and visit notes and viewing radiology results functions with quality measures related to cancer and women’s health [22].

This study aims to examine the relationship between (a) EHR meaningful use and (b) EHR-computerized capabilities among four screening and preventative-oriented quality measures in an ambulatory healthcare setting.

## Materials and methods

Study design and sample

A quantitative retrospective study using a cross-sectional design was used to explore associations between EHR meaningful use and EHR computerized capabilities among four quality measures in physician offices. Secondary data was obtained from the 2015 and 2016 National Ambulatory Medical Care Survey (NAMCS) [23]. NAMCS is a nationally representative survey of ambulatory visits to office-based physicians in the US. The NAMCS uses weighting to account for sampling, clustering, and non-response. The NAMCS utilizes a multistage probability design that involves probability samples of primary sampling units (PSUs), physician practices within PSUs, and patient visits within practices. NAMCS is conducted by the National Center for Health Statistics and is used to produce national estimates that describe ambulatory medical care services in the US.

The response rate for physicians having at least one sampled visit was 30% for 2015 and 39% for 2016. The total office visits for both years was 41,451 patients [23]. We restricted the analytic sample using the following criteria: (a) fulfilling the inclusion criteria of at least one of the four quality measures, (b) only patients age 18 years or older, (c) using partial or all EHR. Therefore, we excluded visits with no EHR use (paper records) (5,543 visits). A total of 30,787 patient office visits met the criteria. But, each CQM had a unique population with a defined age parameter (Table 1).

**Table 1:** Quality measures description, numerator, denominator (population) criteria, sample size, and percentage of patient office-visit records among ambulatory physician offices ^a ^CQM has two levels: screening and then education for eligible patients BMI: body mass index; CQM: clinical quality measure

Quality Measures	Numerator	Denominator	Sample Size (eligible patients)	Visits With Test n (%)
Obesity Screening and Education ^a^	Patients with documented BMI	All patients 18 years and older	30,787	18,178 (59.0)
	Patients with a documented follow-up plan for weight management	Patients 18 years and older with a BMI of 25 or more	15,394	2,540 (16.5)
Tobacco Use Screening	Patients with documented tobacco use status	All patients 18 years and older	30,787	3,435 (11.2)
Blood Pressure Screening	Patients with documented blood pressure measurement	All patients 18 years and older	30,787	20,122 (65.4)

Dependent variables/quality measures

This study focused on four quality measures to examine associations between EHR meaningful use and quality measures in ambulatory care. Three out of the four quality measures focused on screening and one on education/counseling. Quality measures were inspired by the CQMs used in the CMS Quality Reporting Program for the eligible professional final ruling [24]. We selected proxy quality measures that were most compatible with NAMCS data.

Quality measures focused on three different health problems: (a) Obesity (i) BMI screening, (ii) Obesity education; (b) Blood pressure screening; (c) Tobacco use (see Table 1). All quality measures were dichotomized to indicate whether appropriate care was provided and were tested using a single variable from the survey except for obesity education and blood pressure screening. Blood pressure screening (yes/no) was created depending on whether or not readings were available for both systolic and diastolic blood pressure. Obesity education (yes/no) was created depending on whether or not at least one of the three education/counseling services was delivered (diet/nutrition, exercise, and weight reduction counseling). Obesity screening was measured using BMI; if BMI was recorded in the record - regardless of the measurement.

Independent variables

Electronic Health Records Meaningful Use

The original variable for “EHR meaningful use” was measured using the item “does your current system meet meaningful criteria as defined by the department of health and human services?”, which had five answer categories (blank, don’t know, refuse to answer, yes, and no), which we recoded into dichotomous categories: (a) Yes “met meaningful-use criteria” and (b) No. We grouped “no” with blank, don’t know, refuse to answer, which were considered unknown. 

Electronic Health Records: Computerized Capabilities

We examined 10 EHR-capabilities that are most relevant to the four quality measures. Capabilities were recoded as yes/no, with “unknown” being included as “no.” Likewise, “yes but turned off or not used” was grouped with “yes.” The rationale for doing that is that the question from the physician induction survey instrument is “Does the reporting location have any of the following computerized capabilities?” is asking if the facility has the computerized capabilities rather than if they are using it. These capabilities included; (a) recording patient history and demographic information; (a) recording patient problem list; (c) recording clinical notes; (d) recording patient’s medications and allergies; (e) reminders for guideline-based interventions and/or screening tests; (f) ordering lab tests; (g) viewing lab results; (h) identifying patients due for preventive or follow-up care in order to send patients reminders; (i) generating lists of patients with particular health conditions; (j) providing patients with clinical summaries for each visit.

Covariates

Patient and visit characteristics included: (a) facility/visit region (Midwest, Northeast, South, West); (b) location (metropolitan, non-metropolitan); (c) payment used for visits (private, Medicare/state or Medicaid, and other-includes unknown); (d) age, originally continuous recoded to 18-34 years, 35-49 years, 50-69 years, 70 years and over; (e) sex (male, female); (f) race (collapsed to white, other), race data was imputed because 26% and 28% were missing in NAMCS 2015 and 2016, respectively; and (g) ethnicity (Hispanic, non-Hispanic).

Statistical analysis

We used descriptive statistics (chi-square) and multivariate logistic regressions to examine associations with quality measures, which included patient and visit characteristics as covariates. Regression analysis was based on subsets of the sample that met inclusion for each quality measure. The sample size was 30,787 office-visits for three quality measures and 15,394 office visits for obesity education.

We conducted two different adjusted logistic regressions (two different sets of independent variables) with each of the four quality measures as the dependent variables. First, EHR meaningful use was the independent variable (four regression models). Second, the 10 EHR-computerized capabilities were used as the independent variables (40 regression models).

All analyses took into consideration the complex survey design and were performed using SAS software version 9.4 (SAS Institute Inc., Cary, North Carolina). This study received institutional review board (IRB) exemption due to the use of de-identified medical records as the main source of data. Also, the IRB at the NCHS recognizes that the analysis of de-identified and publicly available data does not constitute human subjects research as defined in federal regulations and as such does not require IRB review, therefore, approval was not necessary but was sought and granted anyways (IRB# 5190104). 

## Results

This study included 30,787 office-visits, representing an annual estimate of 680 million national office visits to ambulatory office-based physicians. Office visits with all and partial electronic use were stratified by EHR meaningful-use status, of which 95% visits were to offices that achieved EHR meaningful-use criteria and 5% did not.

Table 1 shows the number of visits eligible for each quality measure and the proportion of completed screening or education. The results indicated that screening for tobacco use had the lowest rate out of all quality measures, with only 11.2% of eligible patients received screening. Likewise, only 16.5% of eligible patients received obesity education for weight management. On the other hand, out of all eligible patients, 59% received obesity screening and 65.4% received blood pressure screening (Table 1).

Table 2 shows a comparison of visit characteristics and EHR computerized capabilities between visits to facilities achieving EHR meaningful-use criteria and visits to facilities that did not. Most visits in both categories were for female patients. We also found that the west region had the highest rate of visits to offices that did not achieve EHR meaningful-use criteria (41%) while the south region had the highest rate of visits to offices achieve EHR meaningful-use criteria (36%) (Table 2).

**Table 2:** Weighted office visits' characteristics among ambulatory physician offices NAMCS 2015-2016 (n= 30,787/ N=680,013,900) patient office visits ^a ^Unknown was grouped with other categories; †Unknown was grouped with other categories NAMCS: National Ambulatory Medical Care Survey; EHR: Electronic Health Record

EHR meaningful use	Yes		No/Unknown^ a^	P-value
Total	29,364 (95.4)		1,423 (4.6)	
		%	%	
Age				0.176
18-34 years		16.7	20.9	
35-49 years		18.4	23.5	
50-69 years		39.5	31.7	
70 years and over		25.3	23.9	
Sex				0.800
Male		39.3	38.2	
Female		60.7	61.8	
Ethnicity				0.343
Not Hispanic or Latino		87.2	82.0	
Hispanic or Latino		12.8	18.0	
Race				0.044
White		69.3	56.8	
Other		30.7	43.2	
Region				0.130
Northeast		19.8	16.2	
Midwest		21.3	8.6	
South		36.1	33.9	
West		22.8	41.2	
Location				0.004
Metropolitan		91.7	98.6	
Non-Metropolitan		8.3	1.3	
Payment for visit				0.449
Medicare/State/Medicaid		41.8	36.8	
Private insurance		46.7	53.0	
Other†		11.5	10.1	
EHR Capabilities (yes, the facility has the capability to)				
Record history and demographics		99.5	87.6	<0.001
Record patient problem list		99.2	86.2	<0.001
Record clinical notes		99.0	86.8	<0.001
Record patient’s medication and allergies		99.5	86.9	<0.001
Reminders for interventions/screening		88.0	62.3	<0.001
Order lab tests		86.9	75.4	0.078
View lab results		90.9	76.1	0.007
Identify patients due for preventive or follow-up to send reminders		88.0	60.5	<0.001
Generate lists of patients with particular health conditions		86.3	74.2	0.081
Provide patients with clinical summaries for each visit		93.5	61.2	<0.001

Tables 3-4 present regression models measuring associations between (1) EHR meaningful use, (2) 10 EHR-computerized capabilities, and four quality measures. We found a significant positive association between EHR meaningful use and obesity screening but no association for the remaining three quality measures. We also found two strong significantly positive associations (P-value < 0.001) between (1) EHR computerized capabilities and (2) quality measures (blood pressure screening and obesity screening). Additionally, other significantly positive associations (P-value < 0.005) were found, however, tobacco use screening was not associated with any capability. Each quality measure will be discussed separately.

**Table 3:** Adjusted regression models for EHR meaningful use and EHR computerized capabilities with four quality measures among office visits in ambulatory physician offices Note. EHR meaningful-use and visit characteristic analyses were conducted in one regression. EHR capabilities analyses were done in another regression. ^a^ P-value 0.001; ^b^ P-value 0.05 EHR: Electronic Health Record

Quality Measures	Blood Pressure Screening			Tobacco Use Screening			
	n=30,787			n=30,787			
EHR meaningful use	OR	95% CI	P-value	OR	95% CI	P-value	
No (Ref.)							
Yes	2.12	0.971-4.636	0.0592	1.30	0.871-1.941	0.1996	
Visit characteristics							
Region							
Midwest (Ref.)							
Northeast	0.66^ b^	0.459-0.952	0.0262	0.90	0.714-1.141	0.3910	
South	1.07	0.766-1.509	0.6762	0.88	0.713-1.108	0.2951	
West	0.67^ b^	0.468-0.981	0.0391	0.68^ b^	0.528-0.877	0.0030	
Location							
Metropolitan (Ref.)							
Non-Metropolitan	1.49	0.950-2.349	0.0821	1.83^ a^	1.356-2.474	< .0001>	
Payment for visit							
Private insurance (Ref.)							
Medicare/state-Medicaid	1.10	0.915-1.326	0.3057	1.83^ a^	1.543-2.178	< .0001>	
Other	0.83	0.610-1.136	0.2477	1.88^ a^	1.356-2.474	< .0001>	
Age (years)							
18-34 years	1.16	0.963-1.417	0.1154	0.97	0.807-1.185	0.8195	
35-49 years	1.19^ b^	1.043-1.375	0.0104	1.10	0.930-1.309	0.2582	
50-69 years (Ref.)							
70 years and over	0.83^ b^	0.701-0.996	0.0447	0.27^ a^	0.224-0.334	< .0001>	
Sex							
Male (Ref.)							
Female	1.14^ b^	1.025-1.277	0.0167	0.64^ a^	0.547-0.751	< .0001>	
Race							
White (Ref.)							
Other	1.96^ a^	1.445-2.677	< .0001>	0.91	0.753-1.104	0.3452	
Ethnicity							
Non-Hispanic/Latino (Ref.)							
Hispanic/Latino	0.78	0.506-1.208	0.2672	0.78	0.595-1.037	0.0884	
EHR capabilities (yes, the facility has the capability to)							
No (Ref.)							
Record history and demographics	0.23	0.015-3.726	0.3059	0.52	0.209-1.302	0.1631	
Record patient problem list	5.74^ b^	1.175-28.107	0.0309	2.09	0.724-6.047	0.1724	
Record clinical notes	2.72	0.951-7.821	0.0621	0.87	0.543-1.411	0.5848	
Record patient’s medication and allergies	0.19	0.028-1.315	0.0926	0.82	0.367-1.851	0.6383	
Reminders for interventions/screening	1.51	0.932-2.470	0.0939	1.14	0.837-1.572	0.3925	
Order lab tests	1.91^ b^	1.188-3.093	0.0077	1.05	0.828-1.347	0.6589	
View lab results	3.35^ a^	1.983-5.662	< .0001>	1.21	0.926-1.592	0.1608	
Identify patients due for preventive/follow-up to send reminders	0.93	0.501-1.754	0.8396	1.05	0.808-1.376	0.6953	
Generate lists of patients with particular health conditions	1.16	0.651-2.072	0.6134	1.06	0.821-1.385	0.6308	
Provide patients with clinical summaries for each visit	1.05	0.590-1.899	0.8489	0.95	0.649-1.406	0.8149	

**Table 4:** Adjusted regression models for EHR meaningful use and EHR computerized capabilities with four quality measures among office visits in ambulatory physician offices (Contd.) Note. EHR meaningful-use and visit characteristic analyses were conducted in one regression. EHR capabilities analyses were done in another regression. ^a^ P-value 0.001; ^b^ P-value 0.05 EHR: Electronic Health Record

Quality Measures	Obesity Screening				Obesity Education		
	n=30,787				n=15,394		
EHR meaningful-use		OR	95% CI	P-value	OR	95% CI	P-value
No (Ref.)							
Yes		3.47^ a^	1.742-6.917	0.0004	1.80	0.813-4.016	0.1462
Visit characteristics							
Region							
Midwest (Ref.)							
Northeast		1.01	0.702-1.470	0.9335	1.18	0.762-1.849	0.4488
South		1.29	0.927-1.799	0.1301	1.15	0.748-1.782	0.5153
West		0.81	0.566-1.182	0.2837	0.81	0.508-1.311	0.4010
Location							
Metropolitan (Ref.)							
Non-Metropolitan		1.26	0.852-1.870	0.2446	0.86	0.484-1.538	0.6168
Payment for visit							
Private insurance (Ref.)							
Medicare/state-Medicaid		0.93	0.771-1.123	0.4537	0.96	0.801-1.165	0.7186
Other		0.89	0.645-1.244	0.5119	1.08	0.797-1.481	0.5986
Age (years)							
18-34 years		0.39^ a^	0.314-0.504	< .0001>	0.64^ a^	0.504-0.837	0.0008
35-49 years		0.82^ a^	0.710-0.958	< .0001>	0.95	0.780-1.156	0.6066
50-69 years (Ref.)							
70 years and over		0.75^ a^	0.649-0.879	0.0003	0.83	0.650-1.079	0.1699
Sex							
Male (Ref.)							
Female		0.81^ a^	0.731-0.909	0.0002	1.01	0.867-1.182	0.8807
Race							
White (Ref.)							
Other		1.45^ b^	1.067-1.972	0.0176	1.36^ b^	1.040-1.778	0.0249
Ethnicity							
Non-Hispanic/Latino (Ref.)							
Hispanic/Latino		0.77	0.527-1.152	0.2102	1.33	0.832-2.124	0.2329
EHR capabilities (yes, the facility has the capability to)							
No (Ref.)							
Record history and demographics		0.51	0.076-3.516	0.5010	0.48	0.057-4.178	0.5123
Record patient problem list		6.15^ b^	1.314-28.778	0.0211	1.31	0.220-7.827	0.7657
Record clinical notes		3.42	0.747-15.665	0.1130	1.93^ b^	1.144-3.267	0.0138
Record patient’s medication and allergies		0.11^ b^	0.018-0.673	0.0169	0.28	0.056-1.452	0.1306
Reminders for interventions/screening		1.66^ b^	1.034-2.686	0.0359	1.95	0.973-3.912	0.0598
Order lab tests		1.42	0.916-2.211	0.1161	0.65	0.291-1.479	0.3090
View lab results		2.75^ a^	1.708-4.436	< .0001>	2.37	0.886-6.382	0.0855
Identify patients due for preventive or follow-up to send reminders		0.87	0.534-1.439	0.6023	1.38	0.622-3.095	0.4239
Generate lists of patients with particular health conditions		1.38	0.890-2.166	0.1483	1.11	0.615-2.033	0.7134
Provide patients with clinical summaries for each visit		1.27	0.722-2.240	0.4051	1.16	0.528-2.588	0.7005

Obesity screening

Facilities meeting EHR meaningful-use criteria were more likely to screen for obesity during a visit as compared to facilities not meeting EHR meaningful-use criteria (OR = 3.5, 95% CI [1.742-6.917]). Patients were more likely to have their BMI measured if they were 50-69 years but less likely to get screened if they were females (OR = 0.8, 95% CI [0.731-0.909]). Offices having the capability to view lab results (OR = 2.8, 95% CI [1.708-4.436]), record the patient problem list (OR = 6.2, 95% CI [1.314-28.778]), and have reminders for interventions and screening (OR = 1.7, 95% CI [1.034-2.686]) were also associated with higher odds of BMI measurement (obesity screening).

Obesity education

We did not find a significant association between receiving obesity education among high-risk adults and meeting EHR meaningful-use criteria, However, patients age 18-34 years were less likely to receive obesity education (OR = 0.6, 95% CI [0.504-0.837]) when compared to other age groups and more likely to receive obesity education if they were non-white (OR = 1.4, 95% CI [1.040-1.778]). We also found that offices having the capability to record clinical notes were more likely to give obesity education (OR = 1.9, 95% CI [1.144-3.267]).

Blood pressure screening

We did not find a significant association between blood pressure screening and meeting EHR meaningful-use criteria. However, patients were more likely to have their blood pressure checked if they were non-white (OR = 2.0, 95% CI [1.445-2.677]), females (OR = 1.1, 95% CI [1.025-1.277]). Visit to offices at the West (OR = 0.67, 95% CI [0.468-0.981]) and Northeast (OR = 0.66, 95% CI [0.459-0.952]) were less likely to get screened for blood pressure (OR = 2.0, 95% CI [1.445-2.677]). We also found that offices having the capability to view lab results (OR = 3.4, 95% CI [1.983-5.662]), order lab test (OR = 1.9, 95% CI [1.188-3.093]), and recording the patient problem list (OR = 5.7, 95% CI[1.175-28.107]) were more likely to measure blood pressure at office visits.

Tobacco use screening

We did not find significant associations between meeting EHR meaningful-use criteria or having any EHR computerized capabilities and screening for tobacco use. However, patients were less likely to be screened for tobacco use if they were: 70 years and over (OR = 0.3, 95% CI [0.224-0.334]) or females (OR = 0.6, 95% CI [0.547-0.751]) but more likely to be screened for tobacco use if they used private insurance (OR = 1.8, 95% CI [1.543-2.178]), Medicare/state-Medicaid (OR = 1.9, 95% CI [1.356-2.474]) to pay for their visit and lived in non-metropolitan areas (OR = 1.8, 95% CI [1.356-2.474]).

## Discussion

We found one positive association between EHR meaningful-use and obesity screening but no association for the other three quality measures (obesity education, blood pressure screening, and tobacco use screening). Nonetheless, screening and education were not performed for all eligible patients. While blood pressure and obesity screening had the highest rates, still a large proportion of the patients did not receive screening for these two conditions (35%-41%). Additionally, screening for tobacco use and obesity education rates were very low as seen in Table 1. This might be due to the common processes in place to screen for chronic conditions like blood pressure and obesity.

Previous studies indicated that, in general, clinical quality measures, overall patient care, and health care utilization improved with using EHR systems that fulfill meaningful use [13-15]. Findings from Appari et al., Kern et al., and King et al. are somewhat contradictory to our results since we found limited improvements related to quality measures with EHR meaningful use. We found improvement in one out of four quality measures, namely, obesity screening [13-15]. Confirming our results, Samal et al. (2014) found that EHR meaningful use was associated with higher quality for two out of seven measures, implying that there are limited benefits to using an EHR system that meets meaningful-use criteria on quality measure improvements [11]. Likewise, Jung et al. (2016) indicated little to no difference in quality measures and concluded that participation in EHR meaningful-use had no significant improvements on quality measures [12]. Nevertheless, it is worth noting that in our study, meaningful use was identified for 95% of visits. That may be one of the reasons that earlier studies found a significant association between EHR meaningful-use and quality measures, and we only found a single positive association.

These inconsistences in results may also be attributed to the set of quality measures used. While we found articles examining the association between EHR meaningful-use and quality measures, none of them focused on the same quality measures we studied. We selected proxy quality measures from CMS’s latest reporting criteria. The CMS's updated quality measures according to the different stages of the incentive programs. Previous studies might have used a set of measures that are no longer recommended or were not compatible with NAMCS. Therefore, our results are considered unique and comparisons are limited to general quality measures findings rather than individual measure findings. However, the four quality measures we used have been studied in previous literature with EHR use but not meaningful use while obesity screening and education had inconsistent results. Researchers found the EHR use improved the quality of tobacco prevention and blood pressure screening [16-20]. These findings contradict our results.

We also explored associations between EHR computerized capabilities and four quality measures and found eight significant associations. Offices having the computerized capability to “record patient problem list” and “view lab results” had higher odds of measuring and recording BMI and blood pressure; “Reminders for interventions/screening” had higher odds of measuring and recording BMI; “Order lab results” had higher odds of measuring and recording blood pressure; “Recording clinical notes” had higher odds of delivering obesity education. No EHR capability was associated with screening for tobacco use.

Ancker et al. (2015) results indicated significantly higher performance in quality measures when capabilities were used [16]. Specifically, electronic reminders, order, and view results yielded improved rates of obesity screening, obesity education, screening for tobacco use, and diabetes blood pressure management [16]. For the most part, these findings support our results except for screening for tobacco use since we did not find any significant association for that quality measure. It is worthy to note that Ancker et al. used blood pressure management for diabetics, but we used blood pressure screening. One explanation for the discrepancy is that we conducted a national analysis rather than focusing on a specific geographical area. Another study found that EHR functions have been associated with higher quality for some conditions but not all measures [11,22]. While Samal et al. (2014) and Poon et al. (2010) used a similar set of capabilities, they used a different set of measures than the ones we studied; even for blood pressure, they measured management while we measured blood pressure screening [11,22]. Blood pressure management would require screening for blood pressure, but it includes the delivery of management services.

Our research study has a few limitations. The cross-sectional design of the study prevents inferring causality between variables. Secondary data analysis limited the ability to collect desired attributes and variables. The NAMCS response rate is considered relatively low for both years, which can cause a nonresponse bias. Furthermore, the selected quality measures only represent specific conditions and characteristics of care; therefore, it does not represent the overall care aspect. It is important to note that some associations may be caused by chance alone and some associations are difficult to justify or explain. The perplexing of these associations does not mean they are non-existing. For a few quality measures, the small sample size may have prevented us from finding statistically significant associations. It is noteworthy that less than 5% of visits did not meet EHR meaningful use; this large difference between comparison groups is not ideal.

## Conclusions

We found limited positive associations between EHR meaningful-use and quality measures in ambulatory healthcare. There were little to no differences in quality measures between visits to office-based physicians that achieved meaningful use and those who did not. Yet, we found multiple positive associations between EHR capabilities and quality measures. Having an EHR system with computerized capabilities might improve the likelihood of delivering appropriate care (clinical quality measure). While EHRs offer substantial administrative efficiency over paper records, current patterns of EHR meaningful-use do not always appear to translate into a better quality of care in physician offices. The quality measures used represent specific conditions and not the overall healthcare aspect such as patient health status, mortality, and morbidity.
